# Decreased P2 Waveform Reflects Impaired Brain Executive Function Induced by 12 h of Low Homeostatic Sleep Pressure: Evidence From an Event-Related Potential Study

**DOI:** 10.3389/fnins.2021.599919

**Published:** 2021-03-24

**Authors:** Lingjing Zeng, Haijing Wu, Jialu Li, Haiteng Wang, Songyue Xie, Tianyi Yang, Ziyi Peng, Liwei Zhang, Yongcong Shao, Jing Lv

**Affiliations:** ^1^School of Psychology, Beijing Sport University, Beijing, China; ^2^Sichuan Cancer Hospital and Institute, Sichuan Cancer Center, School of Medicine, University of Electronic Science and Technology of China, Chengdu, China; ^3^Institute of Psychology, Chinese Academy of Sciences, Beijing, China; ^4^The Second Medical Center, Chinese PLA General Hospital, Beijing, China

**Keywords:** sleep pressure, executive function, Go/NoGo, event-related potential, P2

## Abstract

Homeostatic sleep pressure can cause cognitive impairment, in which executive function is the most affected. Previous studies have mainly focused on high homeostatic sleep pressure (long-term sleep deprivation); thus, there is still little related neuro-psycho-physiological evidence based on low homeostatic sleep pressure (12 h of continuous wakefulness) that affects executive function. This study aimed to investigate the impact of lower homeostatic sleep pressure on executive function. Our study included 14 healthy young male participants tested using the Go/NoGo task in normal resting wakefulness (10:00 am) and after low homeostatic sleep pressure (10:00 pm). Behavioral data (response time and accuracy) were collected, and electroencephalogram (EEG) data were recorded simultaneously, using repeated measures analysis of variance for data analysis. Compared with resting wakefulness, the participants’ response time to the Go stimulus was shortened after low homeostatic sleep pressure, and the correct response rate was reduced. Furthermore, the peak amplitude of Go–P2 decreased significantly, and the peak latency did not change significantly. For NoGo stimulation, the peak amplitude of NoGo–P2 decreased significantly (*p* < 0.05), and the peak latency was significantly extended (*p* < 0.05). Thus, the P2 wave is likely related to the attention and visual processing and reflects the early judgment of the perceptual process. Therefore, the peak amplitude of Go–P2 and NoGo–P2 decreased, whereas the peak latency of NoGo–P2 increased, indicating that executive function is impaired after low homeostatic sleep pressure. This study has shown that the P2 wave is a sensitive indicator that reflects the effects of low homeostatic sleep pressure on executive function, and that it is also an important window to observe the effect of homeostatic sleep pressure and circadian rhythm on cognitive function.

## Introduction

Sleep deprivation (SD) has become a common phenomenon in modern society. Reasons for sleep loss include alcohol and coffee intake, technological development, and life stressors. These factors prevent people from getting high-quality sleep. Previous studies have found that sleep loss endangers individual physical and mental health, such as by increasing the risk of cardiovascular disease and obesity (St-Onge and Zuraikat, 2019; Yu et al., 2019). It can also lead to a decline in individual cognitive functions, such as attention, working memory, executive function, and emotional management (Killgore, 2010; Tantawy et al., 2013; Xie et al., 2015; Lo et al., 2016; Krause et al., 2017; Cunningham et al., 2018; Peng et al., 2020), leading to serious traffic accidents. Additionally, studies have shown that lack of sleep can make individual’s neural representation ability unstable (Poh and Chee, 2017). Therefore, studying the effects of SD on individual cognitive function has great applicative value.

Total sleep deprivation (TSD) can also damage multiple cognitive functions, with the most severe impact on executive function (Lim and Dinges, 2010; Lowe et al., 2017). Executive function refers to the cooperative operation of several human cognitive processes; furthermore, it describes the cognitive abilities that control and regulate other abilities and behaviors, including working memory, inhibition control, and task switching (Miyake et al., 2000). Inhibition control is a cognitive process used to stop individuals from showing inappropriately strong responses (Drummond et al., 2006). Inhibition control has been studied in the context of many practical applications, such as psychiatric characteristics (Paiva et al., 2020), weight loss and calorie intake (Carbine et al., 2020), and cigarette dependence (Gantiva et al., 2020), etc. These studies all use Go/NoGo as the measurement paradigm to reflect individual inhibition control behavior through the changes of the specific event-related potential (ERP) components in the brain. The Go/NoGo paradigm reflects the processing of reaction inhibition and is the most widely used paradigm to measure reaction inhibition in the laboratory environment. These studies use electroencephalogram (EEG) to explore the neurophysiological characteristics related to inhibition control in the brain. For example, Gantiva et al. (2020) analyzed the N2 component of ERP and behavioral measurement in the Go/NoGo task and found that graphic health warnings increased the amplitude of the N200 potential, especially graphic health warnings that covered 60% of the front of cigarette packs. They suggested that graphic health warnings increased inhibitory control in adolescents, especially when the graphic health warnings covered 60% of the front of the cigarette pack. There are other studies using the N1, P2, N2, and P3 components to explore the relationship between inhibition and control in people with test anxiety disorder (Zhang W. et al., 2019). In short, neurophysiological studies related to inhibition and control are now very extensive. Moreover, inhibition control involves two cognitive components: attention to incoming stimuli and prevention of automatic response (Lezak et al., 2004). Automatic response refers to a reduced need for attention and cognitive control due to extensive practice in cognitive or motor tasks. In experiments related to behavior inhibition, automatic response can be explored as an auxiliary task, such as the Go stimulation task in the Go/NoGo task. Schneider and Shiffrin (1977) found that a consistent stimulus–response profile can lead to learning and developing an automatic response. In a study conducted with professional typists, Logan (1982) showed that the automatic response may be highly controllable. Moreover, Kusztor et al. (2019) showed that for repetitive tasks, SD will cause serious damage to continuous attention behavior indicators and ERP components; however, it has no significant effect on automatic response. Studies have also indicated that the brain regions involved in the automatic monitoring of cognitive control seem to be less susceptible to the harmful effects of SD (Chee and Chuah, 2008; Tagliazucchi and Van Someren, 2017). Therefore, if the intensity and duration of SD do not reach certain levels, the potential changes in the ERP components related to automatic response may not be apparent.

The main purpose of the Go/NoGo paradigm is to explore the participants’ brain area activation in certain specific situations during error processing, inhibition control (Borbely, 1982; Menon et al., 2001; Jin et al., 2015; Zhang W. et al., 2019), and response competition. Furthermore, it is widely used to measure inhibition control. Previous studies on ERPs have shown that during the Go/NoGo task, different ERP components are related to reaction inhibition and false signals in the participants’ brains (Kiefer et al., 1998). Studies based on the neural mechanism of the Go/NoGo task showed that the visual NoGo stimuli usually lead to 200–300 ms of negative components (NoGo–N2) (Falkenstein et al., 2002), which reflects an inhibitory process related to signal detection and discrimination. Nativ et al. (1992) found that the sensory P1–N1 potential is sensitive to the initiation of the Go/NoGo reaction. Studies have also shown that the early ERP components (P1, N1, and P2) usually appear in visual tasks, and that they are controlled by Go/NoGo execution control conditions. The negative components are regulated first, followed by the positive components (Di Russo et al., 2010). The Go/NoGo paradigm focuses on the amplitude reduction of the N2 component and late positive potential (LPP) component under the condition of memory inhibition (Bergström et al., 2007; Depue et al., 2013). In addition, P3 is also a classic component of Go/NoGo. The N2 component is a typical negative wave located in the central prefrontal region 150–400 ms after stimulus presentation, which is related to conflict monitoring and inhibition control (Mecklinger et al., 2009). LPP appeared in the central parietal lobe 400–800 ms after stimulus presentation, which mainly reflected the extraction of scene details (Rugg and Yonelinas, 2003). P3 was a positive component related to inhibitory control (Azizian et al., 2006). Studies have shown that SD impairs Go/NoGo task performance (Doran et al., 2001; Liu et al., 2015), and some studies have also found that SD has a significant negative effect on inhibition control (Drummond et al., 2006; Schapkin et al., 2006; Breimhorst et al., 2008; Mander et al., 2010; Almklov et al., 2015; Lowe et al., 2017).

Additionally, there are some neuroimaging studies related to response inhibition. Previous research has used functional magnetic resonance imaging (fMRI) to explore executive function, and findings have shown that the brain areas involved in response inhibition mainly include the inferior frontal gyrus, superior frontal gyrus, insula, cingulate gyrus, inferior parietal lobule, superior temporal gyrus, middle gyrus, and basal nerve (Niendam et al., 2012; Steele et al., 2013). Additionally, a Go/NoGo task was also used in an fMRI study of the brain regions involved in error processing after inhibition response failure (Kireev et al., 2016). Researchers who used the Go/NoGo task to measure inhibition found that individual inhibition efficiency decreased after SD, as did activation of the ventral prefrontal cortex (PFC) (Chuah et al., 2006). However, measurement of fMRI data at the time level is flawed, and the function of suppressing early and late responses cannot be better demonstrated.

An EEG provides the highest resolution of brain activity in the millisecond range. Many perception and attention processes are considered to run in a period of less than 1 s (Keil et al., 2014). The analysis of EEG in the time domain is called ERP. The major weakness of ERP technology is its low spatial resolution, although its primary advantage lies in its high temporal resolution. It usually refers to locking EEG activity across the average transient time of experimentation and has the ability to analyze the timing of brain events in milliseconds. Furthermore, ERP is easy to match up with the response time during experimentation. Its equipment is relatively simple, and it does not require specific environmental requirements. Therefore, ERP technology is used, with excellent time resolution, to study the Go/NoGo tasks related to SD. Most SD studies related to ERP focus on the late component of ERP. For example, some studies that used ERP technology to explore the impact of SD on executive function successively reported that the peak amplitudes of the N2 and P3 components are deceased and the peak latencies of the N2 and P3 components are prolonged in the post-SD (Cajochen et al., 1999; Qi et al., 2010; Renn and Cote, 2013; Liu et al., 2015; Gosselin et al., 2019) and reduced ERP peak amplitude. Jin et al. (2015) found that TSD induced a dose-dependent functional decline in the response inhibition of NoGo–N2 and NoGo–P3 on PFC activation. Furthermore, the NoGo–P3 amplitudes were decreased, whereas the NoGo–N2 latency was prolonged compared with the baseline. Meanwhile, a study also suggested that the amplitude of Go–P3 appeared as early as 12 h after waking up, which may reflect the effect of task repetition rather than true SD. On the contrary, the amplitude of NoGo–P3 decreased significantly after 24 and 36 h of waking, indicating the true effect of SD (Gosselin et al., 2019). However, some studies have found that the early components of ERP are also affected by SD. Early sensory processing after SD showed a slowdown in the sensory coding process and a delay in the attention process, which was reflected in a decrease in the N1 peak amplitude and an increase in latency (Brauer, 2016). A study showed that the amplitude of the N1–P2 complex decreased upon the first awakening (after 2 h) of the baseline night as compared with pre-sleep wakefulness levels; during the recovery night, the decrease of the N1–P2 amplitude was present also upon the second (after 5 h) and final awakening (after 7.5 h). N1 latency increased upon the two nocturnal awakenings regardless of the night, whereas P2 latency was not affected. Moreover, the N1–P2 amplitude increased during recovery at the frontal midline derivation as compared with baseline, whereas it decreased at Pz and Oz scalp locations (Ferrara et al., 2002). This illustrated that the N1–P2 amplitude, the N1 latency, is sensitive in showing a state of brain deactivation during the sleep–wake transition. Gorgoni et al. (2014) have found the following: a post-TSD amplitude increase of the P14 was observed in the Cp3 and C3 scalp locations; the amplitude of the P25 increased at all the three scalp locations under consideration (Cp3, C3, and P3); the N30 shows an amplitude increment in the P3 derivation alone; and, amplitude enhancement of the P40 was found at the Cp3 and C3 scalp locations after SD. Others also researched the event-related EEG parameters that decreased throughout SD. Specifically, the ERP component P1 diminished in amplitude after 24 h of TSD. It is suggested that ERP measures (such as the amplitude of the P1) serve as a complimentary method to track the deterioration of attention and performance during sleep loss (Hoedlmoser et al., 2010). Corsicabrera et al. (1999) found that after 40 h of TSD, processing related to visual tasks showed reduced peak amplitude and prolonged latency on the ERP components of 140–288 ms. Furthermore, some studies have shown that SD can reduce the peak amplitude and prolong the latency of the early ERP components (about 160–200 ms) (Gunter et al., 1987; Humphrey et al., 1994; Lorist et al., 1994; Smith et al., 2002). However, the above studies have not yet reached a consensus regarding the damage SD inflicts on early cognitive processes. Smith et al. (2002) posited that the P2 waves were sensitive to changes in task attention; however, there are few EEG studies on the P2 waves related to SD (Mograss et al., 2009). The P2 wave appears in the early stages of information processing, reflecting the perception of object shapes. It is related to the early recognition of target stimuli. However, while the P2 component is still controversial in some cognitive processes, we can contend that the P2 component reflects the attention and visual processing, and that it is usually considered to be related to selective attention and working memory, reflecting the early judgment of the perceptual process (Saito et al., 2001). Studies have shown that SD can significantly reduce the P2 amplitude in working memory and increase its latency. Moreover, most studies have focused on long-term SD; therefore, whether low homeostatic sleep pressure has an adverse effect on individual cognitive processing and changes in the early ERP components has not yet been systematically concluded. Furthermore, there have been inconsistencies among some research findings (Evans and Federmeier, 2007; Wiggins et al., 2018; Zhang L. et al., 2019).

While the adverse effects of SD are fairly well known, the consensus is that SD does serious damage to cognitive and executive function. Sleep pressure rises during waking, declines during sleep, and increases with SD. Therefore, we can consider 24, 36 h, or even a longer period of SD as high homeostatic sleep pressure, but as for 12 h of continuous wakefulness, we called it as low homeostatic sleep pressure. However, by the time the adverse effects of high homeostatic sleep pressure are identified, it has already become serious. To prevent the impact of high homeostatic sleep pressure on people, we hope to know exactly how early such damage begins to appear, and we aim to explore when the changes to the ERP components occur at various stages of low homeostatic sleep pressure. In fact, high homeostatic sleep pressure includes the effect of SD and the circadian process. Borbely (1982) thought that both the circadian process and sleep homeostasis participate almost equally in the two-process model of SD. However, since the awake time of low homeostatic sleep pressure is not so long, and specially, since this study focuses on the time of low homeostatic sleep pressure, it is more representative of the circadian process. Therefore, in this study, we used a visual Go/NoGo task to simultaneously record the participants’ low homeostatic sleep pressure EEGs to explore the impact of low homeostatic sleep pressure on executive function. The research focused on the following: (1) the dynamic changes of the early ERP components in the Go/NoGo task after low homeostatic sleep pressure and (2) the discovery of a special time window that reflects damage to executive function in the low homeostatic sleep pressure stage. We hypothesized that the P2 wave in the Go/NoGo task would be affected by low homeostatic sleep pressure, which would then impair executive function.

## Materials and Methods

### Participants

This experiment included 14 healthy male participants, aged 18–28 years (25.9 ± 2.3). Previously, these data were used in a visual ERP study of response inhibition in SD (Jin et al., 2015). The participants all had good sleep habits (Pittsburg Sleep Quality Index score <5), were right-handed, and did not have any mental or physical illnesses. In the Raven test, all participants had normal or corrected-to-normal vision above 1.0 and an intelligence score >110. Before the start of the experiment, the experimenter explained the study and relevant precautions to the participants to ensure that they were are familiar with the methods and procedures. In the first 2 weeks of the experiment, the participants slept regularly for 7–9 h/day, and they did not smoke, drink coffee or alcohol, or take any drugs in the first 2 days of the experiment. Before the experiment, all participants provided written informed consent, and the experimental protocol was approved by the Research Ethics Committee of Beijing Military General Hospital.

### Experimental Design

This experiment used a visual Go/NoGo test to evaluate the participants’ executive control function. The arrows were pointing to the left and right directions randomly in the test, with a stimulus signal of 2.0 cm × 0.5 cm (width: 1.5°, height: 0.4°). The tasks were presented in two parallel tests, each with 200 trials (Go stimuli accounted for 2/3 of the total stimuli and NoGo stimuli accounted for 1/3 of the total stimuli). The presentation time for each stimulus was 80 ms, and the interval between two adjacent stimuli was 800 ms.

**FIGURE 1:**
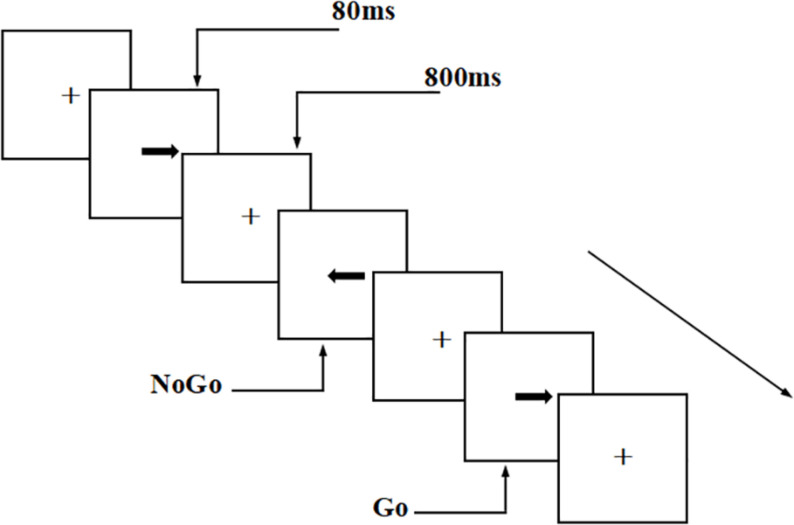
Schematic diagram of visual Go/NoGo test. The tasks were presented in two parallel tests, each with 200 trials (Go stimuli accounted for 2/3 of the total stimuli and NoGo stimuli accounted for 1/3 of the total stimuli). The presentation time for each stimulus was 80 ms, and the interval between two adjacent stimuli was 800 ms.

In the first test, the participants were asked to respond to the “right” arrow, which was the Go stimulus, by pressing the “right button” on the keyboard; they were asked to give no response to the NoGo stimulus. The second test was a balance test, with the opposite stimulus types (i.e., NoGo stimulus: “right” arrow; Go stimulus: “left” arrow). The participants needed to respond to the Go stimulus as soon as possible, but not the NoGo stimulus. The experiment was conducted in a quiet, magnetically shielded, dark room. EEG recording was performed during the visual Go/NoGo task. The participants were required to maintain a fixed gaze (visual angle, 3.29 × 1.76°) throughout the experiment. The participants conducted practice tests before the formal test to ensure that they understood the task. All participants had accuracy rates of over 90% in the practice test. Throughout the test, the participants were asked to respond as quickly as possible, under the premise of ensuring accuracy (Figure 1).

### Experimental Procedures

This study adopted a pre-test to post-test design, and each experiment had two participants at the same time. Before the start of the formal test, the participants performed Go/NoGo test exercises to familiarize themselves with the experimental procedures and laboratory environment, and the practice accuracy rate in each test would reach 90%.

After 1 week of normal sleep, the subjects visited the laboratory for the formal experiment. They had been required to maintain a regular sleep schedule of 8 h per night at least 1 week before the study. Furthermore, all participants were asked to maintain a sleep diary before the experiment to ensure sleep quality. In this study, we took the low homeostatic sleep pressure process from the research data of Jin et al. (2015). The participants visited the laboratory the day before the experiment and slept in the laboratory that night. The TSD started at 10:00 am the next day (Day 1) after a routine sleep, and all participants needed to complete four rounds of the Go/NoGo tasks within 12-h intervals. Therefore, in our study, we took the first low homeostatic sleep pressure process, that is, the participants completed the first Go/NoGo task at 10:00 am (pre-test) and then finished the second Go/NoGo task after 12 h (10:00 pm) (post-test). The nursing staff accompanied the participants all the time to prevent the participants from falling asleep throughout the TSD session. Throughout the TSD experiment period, the participants were not allowed to smoke or drink tea and beverages containing irritating and excitatory substances, such as doping. The participants were not allowed to engage in intense activities and could not leave the laboratory until the test is finished.

### EEG Recordings

Continuous EEG recordings were made with a 32-channel SynAmps2 amplifier (Compumedics Neuroscan, Charlotte, NC, United States). EEG collection was conducted in a soundproof, dark, and room temperature environment of 23°C. The experiment used the Ag/AgCl 64 conductive electrode cap produced by Neuroscan Company, and the electrodes were located at 32 positions as designated by the international 10–20 system. A single recording electrode was placed on each of the participants’ bilateral mastoid processes. With the bilateral mastoid processes (M1 and M2) as reference electrodes, the forehead was used as a ground, and two electrodes were placed above and below the left eye socket to record horizontal electrooculogram (HEOG). Electrodes were placed 1.5 cm outside the left and right corners of the eye to record the vertical electrooculogram (VEOG) to remove interference. The impedance of each electrode point was lower than 5 KΩ, and the band-pass filters were applied between 0.05 and 100 Hz with a modest 12/24-dB slope during continuous recording, which included a 50 Hz notch filter to remove power supply noise. EEG recordings were sampled at 1,000 Hz.

The EEG recordings were processed with the EEGLAB (version 14.1.1b) and ERPLAB toolboxes within Matlab R2019a. The first step was to merge behavioral data, then use EEGLAB to preprocess EEG data, including EEG preview, manually remove data with obvious drift or artifacts, epochs ranging from −100 to 800 ms of the continuous EEG data were extracted and filtered with an Infinite Impulse Response (IIR) Butterworth filter with a passband of 0.5–30 Hz with a frequency slope of 24 dB/oct, and perform independent component analysis (ICA) on the data. ICA is an important development of statistical signal processing. Data decomposition by the ICA method is based on the spatial transformation of “virtual channel” to analyze the linear changes of data from a single scalp channel. It can decompose the signal into several independent components, which can effectively identify and remove the artifacts without knowing the process of generating the artifacts. After ICA, blind source separation is used to separate the source signal and noise interference. Next, ADJUST1.1.1 plug-in within EEGLAB was used to semi-automatically remove independent components with obvious artifacts, and then ERPLAB was used to perform EEG analysis on the data after removing the artifacts. The baseline was corrected to a mean amplitude of 100 ms before the stimulus. The trials in which the voltage exceeded ± 100 μV were rejected automatically by the system. The components were calculated with only the corrected responses, and the main ERP components were averaged. The grand-averaged ERPs were displayed graphically in order to identify the major peaks. The Go–N1, Go–P1, Go–P2, NoGo–N1, NoGo–P1, and NoGo–P2 components were defined as the maximum negative or positive peaks within the 50–130 (P1 component), 70–200 (N1 component), and 130–300 ms (P2 component) latency windows, respectively. Only the components that were recorded at the F3, Fz, F4, C3, Cz, C4, P3, Pz, and P4 electrodes were included for further analysis.

### Data Analysis of Behavioral Experiments

Due to technical errors, data from two participants were excluded; the remaining 12 participants were included in the statistical analysis described below. Behavioral data included average reaction time and accuracy. The behavioral data under the baseline and low homeostatic sleep pressure state were recorded. The analyses were run by IBM SPSS (V22.2), where the repeated measures analysis of variance (ANOVA) method with Greenhouse-Geisser as Bonferroni *post-hoc* analysis were launched. The statistical behavioral result variables included the means and standard deviations of the Go stimulus response when the participant completed the Go/NoGo test before and after low homeostatic sleep pressure and the mean and standard deviation of the accuracy rate of response to the Go stimulus. The behavior indexes are presented in Table 1.

**TABLE 1:** Performance data (mean ± SD) on the Go/NoGo task in the pre-test and post-test.

	Pre-test	Post-test
Mean reaction time (ms)	293.75 (25.28)	287.01 (27.45)
Correct rate	0.94 (0.05)	0.92 (0.09)

### EEG Data Analysis

**FIGURE 2:**
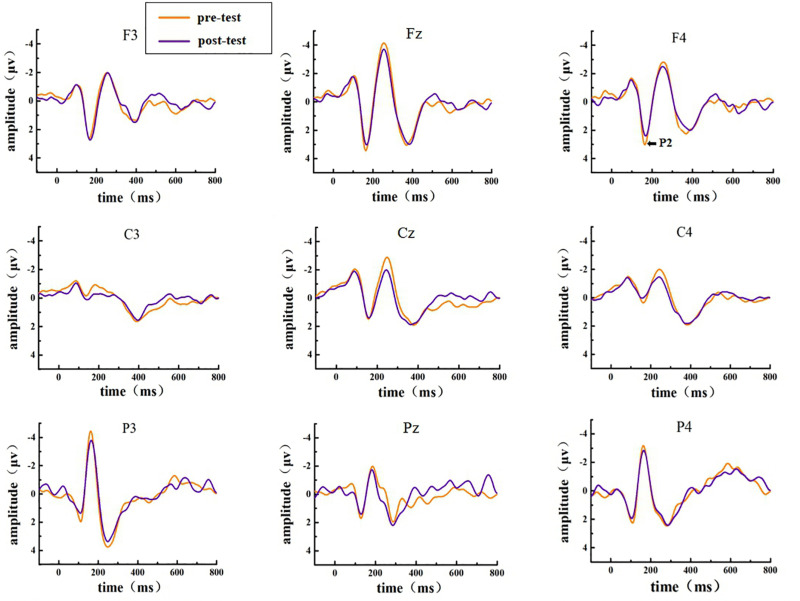
ERP amplitude in the pre-test and post-test for the correct response condition for the Go task. The channels are ordered from left to right and top to bottom as follows: F3, Fz, F4, C3, Cz, C4, P3, Pz, and P4.

**FIGURE 3:**
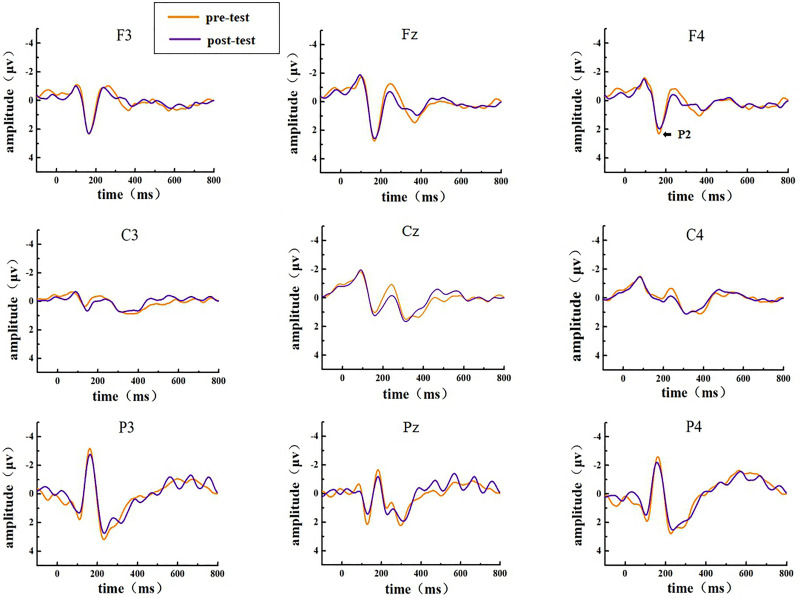
ERP amplitude in the pre-test and post-test for the correct response condition for the NoGo task. The channels are ordered from left to right and top to bottom as follows: F3, Fz, F4, C3, Cz, C4, P3, Pz, and P4.

The EEG activities that responded correctly under the Go and NoGo stimuli were superimposed and averaged; finally, two types of ERP data, Go-correct and NoGo-correct, were obtained. To investigate the time characteristics of the participants’ executive function during the low homeostatic sleep pressure, we first analyzed the ERP component induced by the Go/NoGo task and then used the stimulus presentation as a reference point to measure the average peak amplitude of the components of P1 (50–130 ms), N1 (70–200 ms), and P2 (130–300 ms), which were recorded at F3, Fz, F4, C3, Cz, C4, P3, Pz, and P4 electrodes. The P1, N1, and P2 amplitudes and latencies that were elicited by Go and NoGo trials at the nine electrode sites are presented in Figure 2 (Go task) and Figure 3 (NoGo task).

Repeated measures ANOVA was employed for all ERP results. The main effects and the interactions between sleep states (pre-test and post-test), tasks (Go task and NoGo task), regions (frontal, central, and parietal), and sites [left (F3, C3, P3), middle (Fz, Cz, Pz), and right (F4, C4, P4)] were statistically analyzed employing repeated measures ANOVA, which included Greenhouse–Geisser corrections for non-sphericity and Bonferroni *post hoc* tests. The statistically significant value was set at 0.05 for behavioral index.

## Results

### Behavioral Performance

Compared with the pre-test, the response time for the Go stimulus did not significantly change (293.75 vs. 287.01 s; *t* = 1.710, *p* = 0.103), and the accuracy did not change significantly (94.18 vs. 91.60%; *t* = 1.755, *p* = 0.095) in the post-test (Table 1).

### P1 Component

In the post-test, the statistical analysis of P1 peak amplitude and peak latency showed that the main effect of the peak latency at the measurement time of Go–P1 was not statistically significant [*F*_(1,25)_ = 0.055, *p* = 0.817], and that the main effect of the peak amplitude at the measurement time was not statistically significant [*F*_(1,25)_ = 0.446, *p* = 0.510] in the Go task; the main effect of the peak latency of NoGo–P1 at the measurement time was not statistically significant [*F*_(1,25)_ = 0.117, *p* = 0.736], and its peak amplitude at the measurement time was statistically significant [*F*_(1,25)_ = 0.229, *p* = 0.637] in the NoGo task compared with the pre-test. Moreover, the P1 component showed no significant change in the distribution of scalp voltage.

### N1 Component

In the post-test, the statistical analysis of N1 peak amplitude and peak latency found that the main effect of Go–N1 peak latency at the measurement time was not statistically significant [*F*_(1,25)_ = 0.527, *p* = 0.475], and that the main effect of its peak amplitude at the measurement time was statistically significant [*F*_(1,25)_ = 3.662, *p* = 0.067] in the Go task; the main effect of the peak latency of NoGo–N1 at the measurement time was not statistically significant [*F*_(1,25)_ = 2.151, *p* = 0.155], and its peak amplitude at the measurement time was not statistically significant [*F*_(1,25)_ = 1.076, *p* = 0.310] in the NoGo task compared with the pre-test. Moreover, the N1 component showed no significant change in the distribution of scalp voltage.

### P2 Component

**FIGURE 4:**
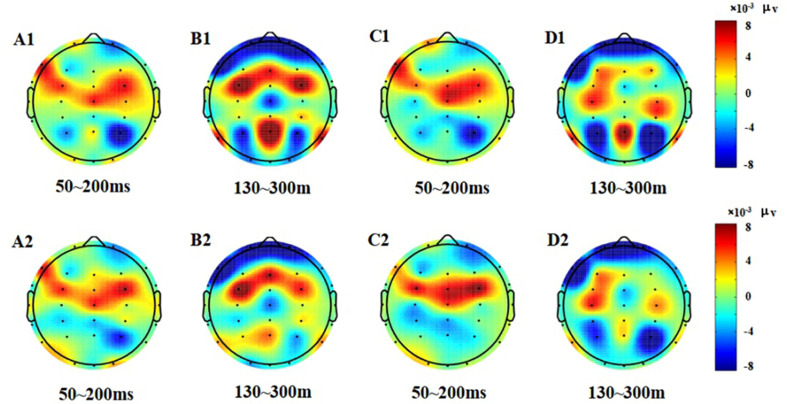
Topographic map of the correct response in the Go/NoGo task **(A1–D2)**. **(A1)** N1, P1, Go stimulation, 50–200 ms, pre-test. **(B1)** P2, Go stimulation, 130–300 ms, pre-test. **(C1)** N1, P1, NoGo stimulation, 50–200 ms, pre-test. **(D1)** P2, NoGo stimulation, 130–300 ms, pre-test. **(A2)** N1, P1, Go stimulation, 50–200 ms, post-test. **(B2)** P2, Go stimulation, 130–300 ms, post-test. **(C2)** N1, P1, NoGo stimulation, 50–200 ms, post-test. **(D2)** P2, NoGo stimulation, 130–300 ms, post-test.

In the post-test, the statistical analysis of P2 peak amplitude and peak latency found that the main effect of Go–P2 peak latency at the measurement time was not statistically significant [*F*_(1,25)_ = 2.712, *p* = 0.112, see Table 4], but its peak amplitude was statistically significant in the main effect of measurement time. In terms of measurement time, the peak amplitude of Go–P2 compared with the baseline value decreased in the post-test [*F*_(1,25)_ = 12.878, *p* < 0.01, see Table 2], and the Go–P2 component appeared on the Fz and F4 electrodes in the Go task. The main effect of NoGo–P2 peak amplitude and peak latency at the measurement time level was statistically significant. At the measurement time level, the peak amplitude of NoGo–P2 decreased after 12 h of low sleep pressure [*F*_(1,25)_ = 4.408, *p* < 0.05, see Table 3]. The NoGo–P2 component appeared most obviously on the F4 electrode (Figure 3). Compared with the pre-test, the peak latency of NoGo–P2 was prolonged after 12 h of low sleep pressure [*F*_(1,25)_ = 5.118, *p* < 0.05, see Table 5] in the NoGo task compared with the pre-test (Figure 4). Moreover, the amplitude in the prefrontal electrode sites was significantly different (*p* < 0.01) in the post-test and showed a decrease in amplitude (Tables 2, 3).

**TABLE 2:** Grand-average peak amplitude of the P1, N1, and P2 components in the Go task across multiple electrode sites in the pre-test and post-test.

		Pre-test			Post-test		
		P1	N1	P2	P1	N1	P2
F3	M (SD)	−0.20 (0.64)	−1.14 (1.37)	2.85 (1.50)	0.33 (0.93)	−1.10 (1.11)	2.88 (1.64)
FZ	M (SD)	−0.61 (0.68)	−1.89 (1.64)	3.48 (1.71)	−0.11 (1.14)	−1.77 (1.44)	3.08 (1.55)
F4	M (SD)	−0.10 (0.65)	−1.66 (1.31)	3.35 (1.78)	0.00 (0.97)	−1.57 (1.12)	2.54 (1.38)
C3	M (SD)	−1.01 (0.60)	−1.20 (0.46)	−0.16 (1.20)	−0.62 (0.92)	−1.04 (0.49)	0.30 (1.53)
CZ	M (SD)	−1.97 (1.94)	−2.05 (0.82)	1.52 (1.51)	−1.72 (1.95)	−1.90 (0.59)	1.45 (1.01)
C4	M (SD)	−1.17 (1.03)	−1.49 (0.52)	0.37 (0.93)	−1.25 (0.83)	−1.42 (0.49)	0.04 (0.96)
P3	M (SD)	1.96 (1.49)	−4.44 (2.70)	3.75 (2.39)	1.36 (1.36)	−3.79 (2.44)	3.36 (2.45)
PZ	M (SD)	1.69 (1.34)	−1.98 (1.13)	1.99 (1.99)	1.41 (1.00)	−1.73 (0.89)	2.20 (1.64)
P4	M (SD)	2.25 (1.36)	−3.17 (2.64)	3.42 (1.43)	1.94 (1.58)	−2.83 (2.95)	2.42 (1.40)

**TABLE 3:** Grand-average peak amplitude of the P1, N1, and P2 components in the NoGo task across multiple electrode sites in the pre-test and post-test.

		Pre-test			Post-test		
		P1	N1	P2	P1	N1	P2
F3	M (SD)	−0.23 (0.58)	−1.01 (1.33)	2.57 (1.43)	0.08 (0.62)	−0.91 (1.06)	2.72 (1.42)
FZ	M (SD)	−0.64 (0.68)	−1.44 (1.42)	3.04 (1.59)	−0.14 (1.07)	−1.54 (1.06)	2.83 (1.93)
F4	M (SD)	−0.58 (0.39)	−1.45 (1.17)	2.98 (1.83)	−0.14 (0.93)	−1.48 (0.76)	2.05 (1.49)
C3	M (SD)	−0.65 (0.69)	−0.66 (0.36)	0.86 (1.12)	−0.48 (0.99)	−0.67 (0.44)	1.04 (1.13)
CZ	M (SD)	−1.31 (1.66)	−1.83 (0.72)	1.32 (1.15)	−1.64 (1.78)	−1.95 (0.74)	1.51 (1.12)
C4	M (SD)	−1.16 (0.86)	−1.15 (0.47)	0.37 (0.98)	−1.20 (0.92)	−1.47 (0.49)	0.62 (1.16)
P3	M (SD)	−1.79 (1.36)	−3.15 (1.97)	3.18 (2.14)	1.32 (1.34)	−2.76 (2.39)	2.74 (1.94)
PZ	M (SD)	2.15 (1.07)	−1.65 (0.85)	1.02 (1.55)	1.43 (0.83)	−1.18 (0.63)	1.47 (1.21)
P4	M (SD)	1.92 (1.28)	−2.60 (2.08)	2.78 (1.35)	1.48 (1.27)	−2.22 (2.31)	2.52 (1.24)

**TABLE 4:** Grand-average peak latency of the P1, N1, and P2 components in the Go task across multiple electrode sites in the pre-test and post-test.

		Pre-test			Post-test		
		P1	N1	P2	P1	N1	P2
F3	M (SD)	66.07 (13.81)	104.57 (11.18)	163.18 (11.22)	66.64 (13.78)	99.00 (7.49)	173.64 (16.11)
FZ	M (SD)	65.79 (14.68)	104.14 (11.20)	165.00 (9.94)	68.11 (14.18)	98.29 (8.60)	171.18 (13.49)
F4	M (SD)	63.14 (13.97)	100.00 (10.10)	166.25 (10.10)	68.11 (13.05)	98.74 (7.91)	176.89 (22.86)
C3	M (SD)	63.79 (16.04)	84.52 (12.90)	138.50 (35.20)	63.80 (12.27)	86.59 (15.79)	142.36 (25.33)
CZ	M (SD)	83.00 (7.73)	90.36 (12.39)	159.14 (26.06)	78.25 (9.22)	88.20 (13.08)	160.29 (24.32)
C4	M (SD)	77.15 (12.59)	88.28 (20.01)	162.89 (25.97)	69.95 (11.79)	84.54 (18.32)	159.50 (24.57)
P3	M (SD)	111.78 (14.38)	161.71 (35.19)	247.64 (23.47)	108.92 (15.77)	164.68 (28.30)	249.93 (20.30)
PZ	M (SD)	128.00 (18.89)	185.25 (18.59)	286.32 (34.80)	127.74 (9.86)	182.21 (21.28)	285.71 (34.98)
P4	M (SD)	109.22 (17.80)	162.71 (21.39)	280.04 (23.61)	105.54 (18.55)	164.13 (26.41)	216.18 (29.56)

**TABLE 5:** Grand-average peak latency of the P1, N1, and P2 components in the NoGo task across multiple electrode sites in the pre-test and post-test.

		Pre-test			Post-test		
		P1	N1	P2	P1	N1	P2
F3	M (SD)	72.96 (13.70)	106.61 (11.16)	170.78 (17.57)	66.93 (28.52)	101.11 (9.41)	170.36 (17.10)
FZ	M (SD)	71.29 (13.82)	104.29 (10.74)	171.64 (10.39)	68.68 (29.57)	101.00 (10.73)	175.96 (14.66)
F4	M (SD)	68.64 (12.93)	101.00 (9.99)	170.00 (17.26)	71.89 (33.09)	99.68 (11.01)	173.71 (17.06)
C3	M (SD)	68.93 (16.74)	74.32 (11.38)	136.27 (22.59)	73.18 (33.09)	90.68 (10.92)	148.93 (33.42)
CZ	M (SD)	56.96 (10.36)	87.07 (11.21)	160.89 (30.73)	73.18 (37.38)	89.29 (10.53)	159.46 (30.67)
C4	M (SD)	60.64 (13.58)	82.57 (10.79)	189.39 (30.50)	62.71 (33.53)	80.89 (9.92)	200.79 (29.10)
P3	M (SD)	111.14 (11.49)	160.39 (20.46)	232.93 (17.68)	110.21 (15.30)	163.82 (17.98)	235.71 (19.80)
PZ	M (SD)	129.11 (13.91)	183.68 (19.96)	227.36 (31.20)	130.89 (9.24)	182.46 (22.53)	230.71 (34.06)
P4	M (SD)	107.04 (17.84)	161.00 (16.14)	225.57 (16.36)	104.07 (19.24)	157.71 (20.54)	235.32 (21.14)

## Discussion

This study used ERP technology to explore the impact of low homeostatic sleep pressure on executive function. In the behavioral analysis, we observed that after low homeostatic sleep pressure, the participant response time to the Go stimulus and the response accuracy rate did not significantly change. This may be due to a relatively low homeostatic sleep pressure; thus, the impairment of individual cognitive processes could not be identified clearly from behavioral techniques. Therefore, through ERP technology, we found that the P2 component changes significantly in the low homeostatic sleep pressure. This finding was a meaningful supplement to the current research on sleep loss.

P2 is considered to be an indicator of stimulus classification (Paiva et al., 2016), reflecting the basic processes of attention distribution, perceptual learning, and memory (Arnott et al., 2011). The change to the P2 wave in working memory requirements is also very sensitive (Smith et al., 2002). A study has shown that sleep pressure can significantly reduce the P2 peak amplitude in working memory and increase the latency (Zhang L. et al., 2019). There are two potential reasons for the decrease in P2 peak amplitude: information processing changes and damage to information in early recognition. However, a feature of our study is the focus on low homeostatic sleep pressure, which means the effect of the low homeostatic sleep pressure in executive function processing. We found that after low homeostatic sleep pressure, the peak amplitude of Go–P2 decreased significantly compared with the pre-test, yet the latency did not change significantly. The peak amplitude of NoGo–P2 decreased significantly, and the latency was significantly extended.

The conclusions obtained in this study can also be demonstrated through Zhang L. et al.’s (2019) interpretation. According to Zhang L. et al.’s (2019) findings on NoGo–N2, we can posit that the latency of NoGo–P2 may reflect increases in response time after sleep pressure. The latency of Go–P2 did not change significantly, which also confirmed that low homeostatic sleep pressure did not impact individual automatic response (Chee and Chuah, 2008; Krause et al., 2017; Tagliazucchi and Van Someren, 2017). The P2 peak amplitude was decreased in both the Go and NoGo tests, indicating that individual top-down cognitive control ability was gradually decreased. The P2 wave is related to visual processing and reflects the early judgment of the perceptual process. The decrease in P2 peak amplitude also confirmed that low homeostatic sleep pressure had begun to disrupt the executive function to a certain extent. Because the P2 component is thought to reflect the early judgment of the perceptual and visual processing and the P2 latency is thought to reflect the time window for attention process, the increase in the P2 latency tendency that occurred during the low homeostatic sleep pressure suggested that more time was needed for attention resource allocation after low homeostatic sleep pressure. The decreased amplitudes of the NoGo–P2 potentials after low homeostatic sleep pressure, which may reflect the subjects’ reduced visual processing, also showed reduced judgment to the target stimuli. In this study, although no significant changes in the response time and accuracy of the Go test were obtained, the accuracy did have a downward trend.

We speculate that this impairment of executive function may be caused by an individual’s fatigue from working continuously for 12 h (10:00 am–10:00 pm). This fatigue damages an individual’s cognitive resources and reflects changes in the P2 wave. It is worth mentioning that functional compensation is one of the unique functions of the human brain and an important factor in maintaining cognitive function. However, we still found that the peak amplitude of NoGo–P2 was decreased in this study. Previous studies have shown that if the time of sleep pressure is not long enough, the peak amplitude of NoGo–N2 will not change, due to the compensatory response of the brain to increased monitoring requirements (Drummond et al., 2000). Thus, we can argue that, due to the low homeostatic sleep pressure in this study, although the brain’s cognitive function has begun to suffer damage, this damage was not enough to allow the body to reach a level of compensation; thus, its cognitive compensation resources were not used to compensate this impaired cognitive processing function.

Our research found that after low homeostatic sleep pressure, the peak latency of P1 and N1 did not change significantly. We have two explanations for this. First, P1, N1, and P2 are all early ERP components. The P1 wave is related to the consumption of selective attention and attention resources, whereas the N1 wave reflects qualitative attention or early participation. Although the three are affected by physical characteristics, the P1 and N1 waves appear earlier than the P2 wave; thus, the P1 and N1 waves are more affected by physical stimuli and are therefore more strongly related to the physical characteristics of information processing. After low homeostatic sleep pressure, an individual’s physical processing of external visual information is not significantly affected. Second, this study found that the peak amplitude of P2 was decreased and the latency was prolonged. In contrast, we know that cognitive control and response inhibition, two relatively automatic aspects, are not easily affected by SD (high homeostatic sleep pressure) (Drummond et al., 2000); thus, potential changes of the peak latency in the N1 and P1 waves are not obvious after only low homeostatic sleep pressure.

From the difference of scalp voltage distribution, combined with the analysis of the topographic map, the P2 component was significantly related to low homeostatic sleep pressure changes in the PFC. The PFC is particularly susceptible to high homeostatic sleep pressure in activating executive functions (Drummond et al., 2000); thus, this study found that the frontal lobe changed significantly after executive function was affected too by low homeostatic sleep pressure, which means that extended wakefulness would be harmful to the function of the PFC. The PFC is also important in individual advanced cognitive processing (Hartmann et al., 2015). The PFC plays an important role in cognitive control. Studies have shown that the PFC can bypass top-down cognitive control, allowing individuals to focus on target-related information (Smallwood et al., 2011; Wen et al., 2013), while suppressing non-target-related information. The PFC is very important in the study of executive function. Studies have shown that sleep pressure will reduce the activation of the PFC (Ma et al., 2014). Mander et al. (2010) also measured inhibition using the Go/NoGo task and found that after sleep pressure, individual inhibitory efficiency was decreased, and that the activation intensity of the ventral PFC was also decreased.

The EEG results reflect that the activity of the PFC increases after low homeostatic sleep pressure in the 130–300 ms period, indicating that the PFC had begun to compensate for the damage control function at this time; however, the frontal lobe area was not significantly changed in the 50–130 and 70–200 ms periods. Therefore, we believe that the compensatory function of PFC control may be more reflected in the late ERP components of the P2 wave, such as the N2 and P3 waves. Among these classic early and late ERP components, only the P2 wave can reflect the early damage to executive function from low homeostatic sleep pressure.

In the Go/NoGo test of other high homeostatic sleep pressure studies in general, they have successively reported reduced peak amplitude of the N2 and P3 waves and extension of the latency (Lee et al., 2003; Qi et al., 2010; Renn and Cote, 2013; Jin et al., 2015; Liu et al., 2015). Our research did not explore the changes in the P3 components. This is because P3 belongs to the late components of ERP and is considered to reflect the allocation of attention resources. This study focused on the exploration of executive function and information processing. The P3 component is considered to reflect the window of stimulus classification and evaluation time and has specific functional significance. Therefore, exploring the development and changes of P3 is not significant for the early executive control processing explored in this research, and we aimed to explore the change of the early components in the low homeostatic sleep pressure.

Based on the findings of this study, we can speculate that after low homeostatic sleep pressure, executive function reflected on the late components, such as N2 and P3, may also have obvious changes as early processing damage may have a certain effect on late processing. However, there is another possibility that N2 and P3 did not change significantly in the experiment of only low homeostatic sleep pressure. We can explain this because the individuals had corresponding functional compensation during cognitive processing, such as the compensation of the PFC, which may compensate to a certain extent for the impairment of cognitive processing caused by low homeostatic sleep pressure.

A previous study has mentioned that both the circadian process and sleep homeostasis participate almost equally in the two-process model of SD (Borbely, 1982). The homeostatic process depends on prior sleep and wakefulness. Sleep pressure rises during waking, declines during sleep, and increases with SD. The circadian process provides a wakefulness signal that progressively becomes stronger during daytime hours and dissipates rapidly after the onset of nocturnal melatonin (Dijk et al., 1997). The high homeostatic sleep pressure is affected not only by the long-term lack of sleep but also by the circadian rhythm. However, the interaction between circadian rhythms and homeostasis is unclear. Therefore, we suggest that there might be circadian rhythm under the high homeostatic sleep pressure process, which is an unavoidable factor in Jin et al.’s (2015) study. Furthermore, in this study, we only considered the low homeostatic sleep pressure process, which is better suited to prove the change of the circadian rhythm. Therefore, we can also explain that the circadian rhythm causes a significant change in the P2 component, when individuals perform executive function.

This study explored the impact of low homeostatic sleep pressure on executive function and found that executive function declined in the post-test, which was mainly manifested by the damage of the P2 component in ERP. This study provides ERP evidence, highlights the significance of the P2 wave in fatigue monitoring, and shows that P2 is a sensitive indicator reflecting the influence of executive function as well. Furthermore, the P2 component is an important window for observing the impact of low homeostatic sleep pressure on cognitive function.

### Limitations

There were several limitations to this study. First, this study only investigated young males; thus, these results cannot be generalized to older adults or women. Second, the sample size was very small; therefore, we should be cautious about speculation regarding the meaning of our results. Third, we had no control group in this study. However, for this problem, we conceptualized this study as a pre-test to post-test design. However, to make the experimental results more accurate, we will try to set up a control group in the future, which can improve the corresponding evidence. Fourth, we used an online filter during acquisition. This acquisition filter and the filter applied during pre-processing are causal filters and induce phase shifts during acquisition; therefore, it is advisable to have minimal filtering during acquisition in future studies. Finally, in this study, homeostatic sleep pressure, circadian rhythm, or repeated testing may also have played important roles in evoking the observed differences in the ERP components. Therefore, future studies should further distinguish between the influence of sleep pressure and circadian rhythm and explore the effects of low homeostatic sleep pressure on cognitive function.

## Data Availability Statement

The datasets generated for this study are available on request to the corresponding authors.

## Ethics Statement

The studies involving human participants were reviewed and approved by the Research Ethics Committee of Beijing Military General Hospital. The patients/participants provided their written informed consent to participate in this study.

## Author Contributions

YS and JLv designed the experiments. YS collected the experimental data. LZe and HWu produced the results and wrote the manuscript. JLi, HWa, SX, ZP, LZh, and TY analyzed and interpreted the data. All authors listed have read and approved the final manuscript.

## Conflict of Interest

The authors declare that the research was conducted in the absence of any commercial or financial relationships that could be construed as a potential conflict of interest.
